# Does Robotic Liver Surgery Enhance R0 Results in Liver Malignancies during Minimally Invasive Liver Surgery?—A Systematic Review and Meta-Analysis

**DOI:** 10.3390/cancers14143360

**Published:** 2022-07-11

**Authors:** Mirhasan Rahimli, Aristotelis Perrakis, Mihailo Andric, Jessica Stockheim, Mareike Franz, Joerg Arend, Sara Al-Madhi, Mohammed Abu Hilal, Andrew A. Gumbs, Roland S. Croner

**Affiliations:** 1 Department of General, Visceral, Vascular and Transplant Surgery, University Hospital Magdeburg, Leipziger Str. 44, 39120 Magdeburg, Germany; aristotelis.perrakis@med.ovgu.de (A.P.); mihailo.andric@med.ovgu.de (M.A.); jessica.stockheim@med.ovgu.de (J.S.); mareike.franz@med.ovgu.de (M.F.); joerg.arend@med.ovgu.de (J.A.); sara.al-madhi@med.ovgu.de (S.A.-M.); roland.croner@med.ovgu.de (R.S.C.); 2 Unità Chirurgia Epatobiliopancreatica, Robotica e Mininvasiva, Fondazione Poliambulanza Istituto Ospedaliero, Via Bissolati, 57, 25124 Brescia, Italy; abuhilal9@gmail.com; 3 Department of Surgery, Centre Hospitalier Intercommunal de Poissy/Saint-Germain-en-Laye, 10 Rue du Champ Gaillard, 78300 Poissy, France; aagumbs@gmail.com

**Keywords:** liver surgery, robotic surgery, laparoscopic surgery, hepatectomy, resection margin, meta-analysis

## Abstract

**Simple Summary:**

The resection margin status is one of the most relevant oncological factors in liver cancer surgery. Whether robotic liver surgery enhances R0 results in liver malignancies during minimally invasive liver surgery is not yet completely clear. We conducted a systematic review with meta-analysis to compare robotic and laparoscopic approaches in liver surgery with particular attention to the resection margin status in liver malignancies.

**Abstract:**

Background: Robotic procedures are an integral part of modern liver surgery. However, the advantages of a robotic approach in comparison to the conventional laparoscopic approach are the subject of controversial debate. The aim of this systematic review and meta-analysis is to compare robotic and laparoscopic liver resection with particular attention to the resection margin status in malignant cases. Methods: A systematic literature search was performed using PubMed and Cochrane Library in accordance with the PRISMA guidelines. Only studies comparing robotic and laparoscopic liver resections were considered for this meta-analysis. Furthermore, the rate of the positive resection margin or R0 rate in malignant cases had to be clearly identifiable. We used fixed or random effects models according to heterogeneity. Results: Fourteen studies with a total number of 1530 cases were included in qualitative and quantitative synthesis. Malignancies were identified in 71.1% (n = 1088) of these cases. These included hepatocellular carcinoma, cholangiocarcinoma, colorectal liver metastases and other malignancies of the liver. Positive resection margins were noted in 24 cases (5.3%) in the robotic group and in 54 cases (8.6%) in the laparoscopic group (OR = 0.71; 95% CI (0.42–1.18); *p* = 0.18). Tumor size was significantly larger in the robotic group (MD = 6.92; 95% CI (2.93–10.91); *p* = 0.0007). The operation time was significantly longer in the robotic procedure (MD = 28.12; 95% CI (3.66–52.57); *p* = 0.02). There were no significant differences between the robotic and laparoscopic approaches regarding the intra-operative blood loss, length of hospital stay, overall and severe complications and conversion rate. Conclusion: Our meta-analysis showed no significant difference between the robotic and laparoscopic procedures regarding the resection margin status. Tumor size was significantly larger in the robotic group. However, randomized controlled trials with long-term follow-up are needed to demonstrate the benefits of robotics in liver surgery.

## 1. Introduction

Robotic procedures are an integral part of modern liver surgery [1]. Various meta-analyses confirmed the comparability of robotic and laparoscopic approaches. With regard to the tumor-free resection margin, robotic and laparoscopic liver surgery show similar outcomes [2,3,4]. The resection margin status is one of the most important oncological parameters in liver cancer surgery [5]. A positive resection margin is an independent risk factor for recurrence-free and overall survival in patients with hepatocellular carcinoma or intrahepatic cholangiocarcinoma [6,7]. R1 resections of colorectal liver metastases in patients receiving perioperative chemotherapy were associated with significantly higher rates of intrahepatic and surgical margin recurrence [8].

Due to the earlier adoption of laparoscopic liver surgery, the number of laparoscopic cases is higher in many studies when compared to robotic liver surgery cases. Since a significant number of these surgeries were performed for benign indications, careful differentiation needs to be taken into account during statistical analysis of the R status. Otherwise, this could lead to a falsely lower percentage of positive resection margin rates.

In some studies and meta-analyses, no attention was paid to the accurate separation of the malignant and benign liver lesions when analyzing the R status of the resection margins. This resulted in inaccurate percentages of the R0 or R positive rates [9,10,11,12,13,14]. Moreover, several individual studies showed that robotic liver surgery achieved an R0 resection in 100% of the cases [15,16,17,18,19,20]. This gave us the idea to take a closer look at the previously published literature in order to systematically analyze the potential advantage of robotics with regard to tumor-free resection margins.

Stable three-dimensional visualization, absence of physiological tremor, higher freedom of movement, better ergonomics for the surgeon and the possibility of using a third arm are the advantages of robots compared to conventional laparoscopic surgery [21,22,23]. Perhaps these advantages of robotics are also beneficial in achieving R0 resection. Furthermore, the use of modern tools in minimally invasive liver surgery, such as ICG fluorescence, can be very helpful in the detection of malignant liver lesions, and the resection margins can be determined very precisely in combination with intra-operative ultrasound. The oncological result can be optimized in this way [15,24]. Although laparoscopy has the theoretical advantage of haptics, this limitation in robotic-assisted surgery may be able to be overcome via visual cues [25].

The aim of this systematic review and meta-analysis is to evaluate the influence of robotic liver surgery on the resection margin status in malignant cases compared to the conventional laparoscopic approach.

## 2. Methods

### 2.1. Literature Search Strategy

A systematic literature search was performed using PubMed and Cochrane Library. Two authors (M.R. and R.C.) independently conducted the systematic search of the articles in English since 2010. The research ended on 2 July 2021. In the event of disagreement, the case was discussed with the assistance of the third author (A.P.). The search terms were “laparoscopy”, “laparoscop*”,“laparoscopic surgery”, “robotics”, “robot*”, “robotic surgery”, “hepatectomy”, “liver resection”, “liver surgery” and “hepatic resection.” These terms were used with help of the boolean operators AND/OR in different combinations and partly using Medical Subject Headings (MeSH). We also manually searched the reference lists of recent systematic reviews and eligible articles for potentially relevant studies for this work.

### 2.2. Aim of Study

The primary aim of our meta-analysis was to compare the robotic and laparoscopic procedures with regard to resection margin status after resection of liver malignancies. Secondarily, the perioperative outcomes, such as operation time, intraoperative blood loss, length of hospital stay, tumor size, overall and severe complications and conversion rate, should be analyzed comparatively between robotic and laparoscopic resections of liver lesions, including non-malignant cases.

### 2.3. Inclusion Criteria

Only studies comparing robotic and laparoscopic liver resections were considered for this meta-analysis. Studies had to include an adequate comparative analysis of laparoscopic and robotic procedures. Above all, the analysis and comparison of the resection margin status in both groups had to be available. Furthermore, the article had to deal with malignant liver lesions, or it had to clearly differentiate between malignant and benign cases with the associated rates of the resection margin status. The malignant cases could include hepatocellular carcinoma, cholangiocarcinoma, colorectal liver metastases and other liver malignancies. Only articles in English were considered.

### 2.4. Exclusion Criteria

The studies without information on the resection margin status or without clear differentiation between malignant and benign cases were excluded. As mentioned, articles in any other language without an English version were excluded. Furthermore, letters, editorials, study protocols, review articles and meta-analyses without original data, case reports and studies with total numbers of cases <20 were excluded. Studies with overlapping data were excluded, and those that were more suitable for our meta-analysis (i.e., studies with more detailed information on R status, a higher number of malignant cases and higher study quality) were retained. Hand-assisted cases were excluded from the meta-analysis.

### 2.5. Data Extraction and Quality Assessment

The data were extracted and tabulated, in accordance with inclusion and exclusion criteria: name of first author, year of publication, country where the study was conducted, study design, case number in each of the robotic and laparoscopic groups, number of malignant cases, number of cases with positive resection margins, age and sex of patients, operative time, intra-operative blood loss, length of stay, size of lesion, complications and conversion rate.

The methodological quality of the studies was assessed using the Newcastle–Ottawa scale (NOS) [26]. According to the NOS, points from 0–9 were awarded per study. The studies with scores ≥ 6 were considered to be of high quality.

### 2.6. Statistical Analysis

This systematic review and meta-analysis were carried out in accordance with the PRISMA guidelines (Preferred Reporting Items for Systematic Reviews and Meta-Analyses) and the protocol established by the authors, taking into account the inclusion and exclusion criteria [27]. Continuous and dichotomous data were analyzed using mean differences (MD) and odd ratios (OR), respectively. The Mantel–Haenszel method was applied for dichotomous variables. When reporting the continuous data as the median and range or interquartile range, we used the method described by Lou et al. and Wan et al. to calculate the mean and standard deviation [28,29]. *p*-values of <0.05 were considered to be statistically significant.

The I^2^ statistic was used to estimate statistical heterogeneity. With I^2^ ≥ 50% and a significance level of *p* < 0.05, high heterogeneity was assumed. In this case, we used the random effects model; otherwise, the fixed effects model was used.

We used the RevMan 5.3 software (The Cochrane Collaboration, Oxford, UK) for data analysis.

## 3. Results

### 3.1. Results of the Literature Search

**Figure 1:**
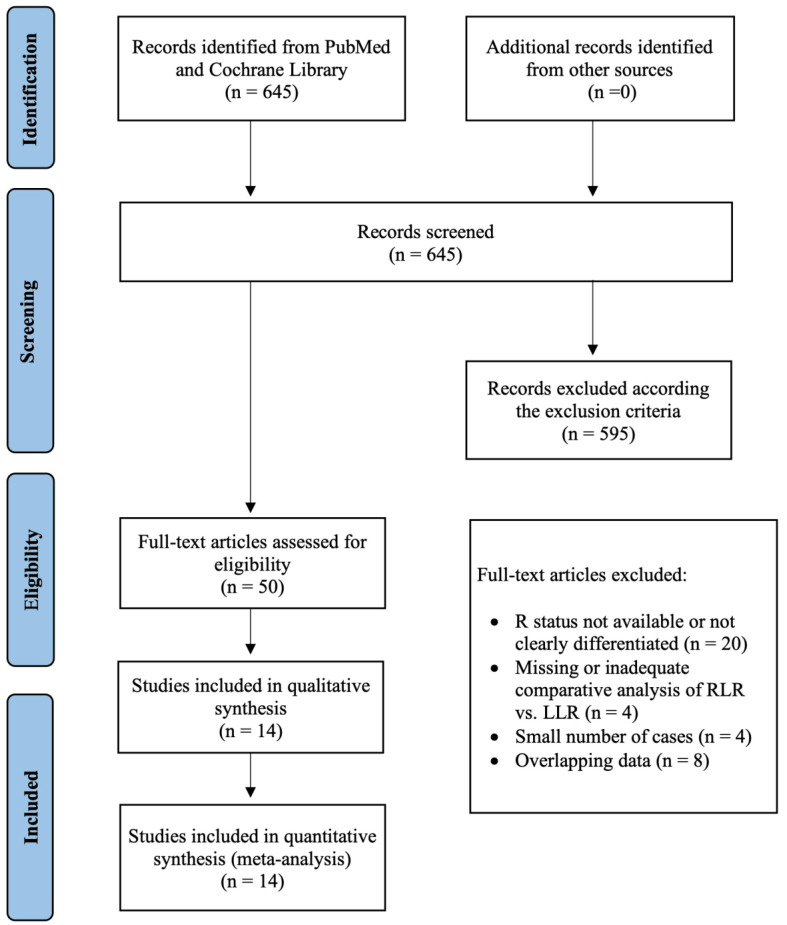
PRISMA flow diagram of the literature research.

A total of 645 records were screened for the inclusion and exclusion criteria. Of these, 50 full-text articles were checked for eligibility. Fourteen of these studies were included in the qualitative and quantitative synthesis (Figure 1). All of them were retrospective in nature. Completed randomized controlled trials were not found. Mejia et al. analyzed and reported the minor and major liver resections separately [30]. Therefore, the data of this study were split accordingly. Table 1 shows characteristics of the included studies. The results of our meta-analysis are summarized in Table 2.

### 3.2. Resection Margin Status

**Figure 2:**
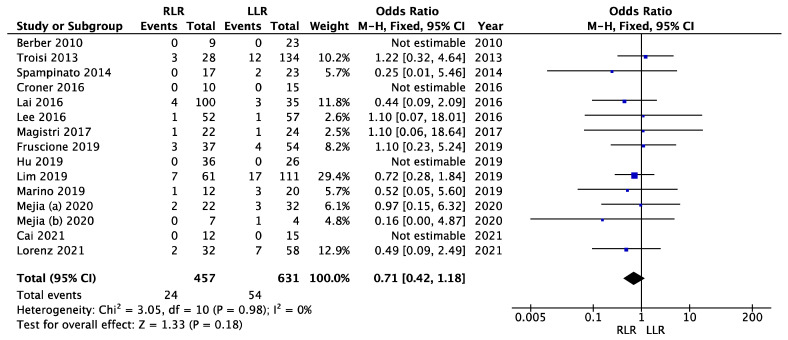
Meta-analysis of positive resection margin status.

In 14 studies, the resection margin status could be clearly assigned to malignant cases. Our meta-analysis included a total of 1530 cases. Of these, 1088 cases (71.1%) were malignancies: 457 cases in the robotic group vs. 631 cases in the laparoscopic group. Figure 2 shows the forest plot of the meta-analysis on positive resection margin status. There was no significant heterogeneity (I^2^ = 0%, *p* = 0.98), so we used the fixed effects model. No significant difference could be shown in the meta-analysis of the positive resection margin status between the robotic and laparoscopic approaches (OR = 0.71; 95% CI (0.42–1.18); *p* = 0.18).

### 3.3. Operation Time

**Figure 3:**
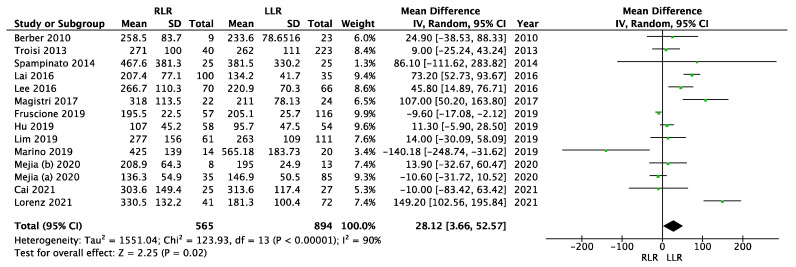
Meta-analysis of operation time.

There was high heterogeneity (I^2^ = 90%, *p* < 0.00001), so we used the random effects model for meta-analysis of operative time (Figure 3). The operative time was significantly higher in the robotic group (MD = 28.12; 95% CI (3.66–52.57); *p* = 0.02).

### 3.4. Intra-Operative Blood Loss

**Figure 4:**
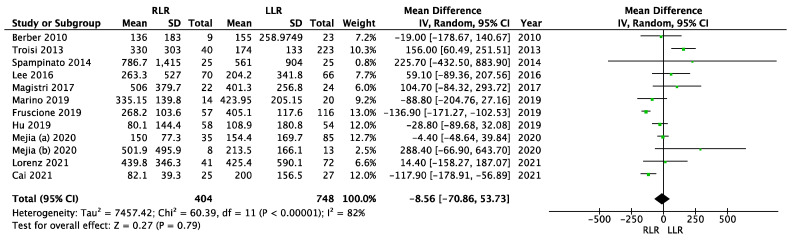
Meta-analysis of intra-operative blood loss.

Thirteen studies reported the intra-operative blood loss, one of them without standard deviation or ranges and another one with mean and range, so these studies were not considered for the meta-analysis of the intra-operative blood loss [33,34]. Lim et al. did not report on intra-operative blood loss [38]. High heterogeneity was observed (I^2^ = 82%, *p* < 0.00001). The random effects model was used (Figure 4). There was no significant difference in intra-operative blood loss between the groups (MD = −8.56; 95% CI (−70.86–53.73); *p* = 0.79).

### 3.5. Length of Hospital Stay

**Figure 5:**
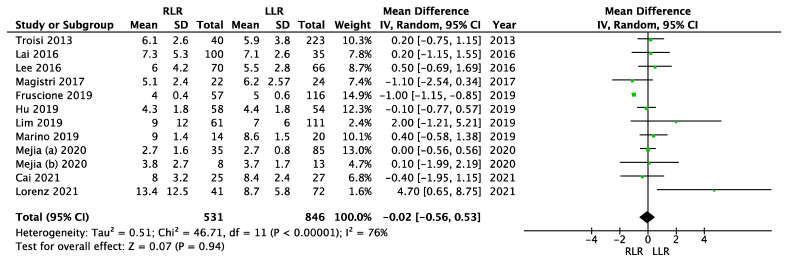
Meta-analysis of length of hospital stay.

The meta-analysis showed no significant difference between the robotic and laparoscopic groups regarding the length of hospital stay (MD = −0.02; 95% CI (−0.56–0.53); *p* = 0.94). We used a random effects model. There was significant heterogeneity (I^2^ = 76%, *p* < 0.00001). Figure 5 shows the meta-analysis of length of hospital stay.

### 3.6. Tumor Size

**Figure 6:**
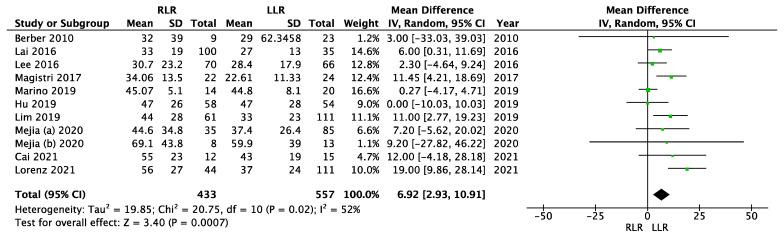
Meta-analysis of tumor size.

Data from ten studies were used for the meta-analysis of tumor size (Figure 6). Two studies did not report the data on tumor size [32,36]. One study presented data as mean and range [33]. In one study, data on tumor size were inconclusive [9]. Therefore, these four studies were excluded from the meta-analysis of tumor size. There was significant heterogeneity (I^2^ = 52%, *p* = 0.02), so we used a random effects model. The meta-analysis showed that the tumor size was significantly larger in the robotic group (MD = 6.92; 95% CI (2.93–10.91); *p* = 0.0007).

### 3.7. Overall Complications

**Figure 7:**
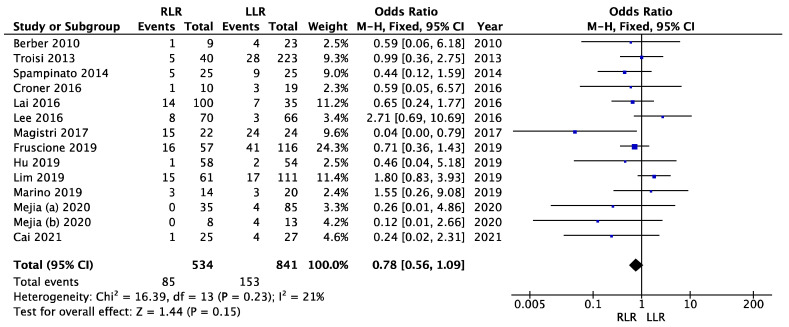
Meta-analysis of overall complications.

All studies reported data on complications. One study reported only the severe complications (Clavien-Dindo grade ≥ 3), so it was excluded from the meta-analysis of overall complications [41]. No significant heterogeneity was observed (I^2^ = 21%, *p* = 0.23). A fixed effects model was used. There was no significant difference between the groups with regard to overall complications (OR = 0.78; 95% CI (0.56–1.09); *p* = 0.15). Figure 7 illustrates the meta-analysis of overall complications.

### 3.8. Severe Complications

**Figure 8:**
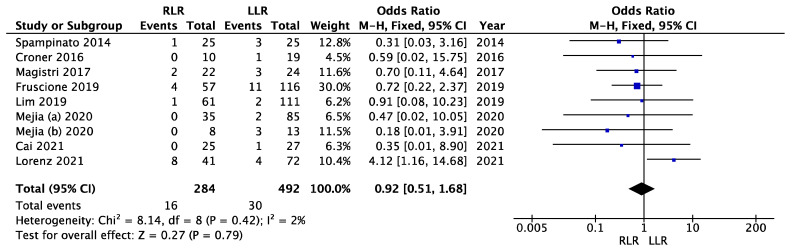
Meta-analysis of severe complications.

In eight studies, severe complications (Clavien-Dindo grade ≥ 3) were reported or could be clearly differentiated (Figure 8). There was no significant heterogeneity (I^2^ = 2%, *p* = 0.42). We used the fixed effects model. The meta-analysis showed no significant difference between the robotic and laparoscopic approaches regarding severe complications (OR = 0.92; 95% CI (0.51–1.68); *p* = 0.79).

### 3.9. Conversion

**Figure 9:**
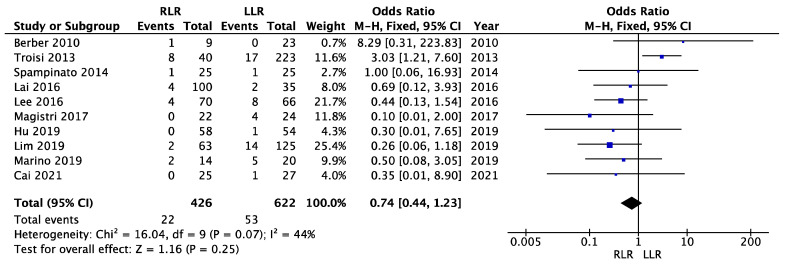
Meta-analysis of conversion.

There was not high heterogeneity regarding the conversion rate (I^2^ = 44%, *p* = 0.07). The fixed effects model was used (Figure 9). There was no significant difference between the groups in terms of conversion rate (OR = 0.74; 95% CI (0.44–1.23); *p* = 0.25).

### 3.10. Liver Malignancies

In our study, 1088 malignant cases were identified. These included 604 hepatocellular carcinomas and 64 cholangiocarcinomas (Table 3). Colorectal liver metastases were detected in at least 305 cases. Fruscione et al. reported liver metastases in 24 cases in the robotic group and 31 cases in the laparoscopic group in their study [36]. It was unclear whether or how many of these were colorectal liver metastases. Cai et al. reported one metastasis in the laparoscopic group in their study [40]. It was also unclear whether it was a colorectal liver metastasis. The remaining malignant cases were other liver malignancies.

## 4. Discussion

Robotic procedures have become indispensable in modern liver surgery. The safety and feasibility of this approach is no longer a topic of discussion. However, the advantages of the robotic approach in comparison to conventional laparoscopic and open procedures in liver surgery are the subject of considerable debate [1]. Except for the longer operative time and higher costs of robotic liver surgery, robotic and laparoscopic approaches to liver surgery have largely similar peri-operative results. There were no significant differences between the two procedures with regard to blood loss, blood transfusion, length of hospital stay, tumor-free resection margin or complication rate in the previous analyses [2,3,4].

In our study, the operation time was significantly longer in the robotic group than in the laparoscopic group (MD = 28.12; 95% CI (3.66–52.57); *p* = 0.02). There were no significant differences between procedures in terms of intra-operative blood loss, length of hospital stay, overall and severe complications or conversion rate.

Our meta-analysis included a total of 1530 cases. Malignancies were identified in 71.1% (n = 1088) of these cases. A positive resection margin was observed in 5.3% of cases (n = 24) in the robotic group and in 8.6% of cases (n = 54) in the laparoscopic group. However, this difference was not statistically significant (OR = 0.71; 95% CI (0.42–1.18); *p* = 0.18). Nevertheless, there was a trend in favor of robotic liver surgery, considering previous analyses. Montalti et al. compared 155 vs. 395 liver resections in robotic and laparoscopic groups, respectively, for resection margin status in their meta-analysis. There were 23 cases (14.8%) in the robotic group and 33 cases (8.4%) in the laparoscopic group with positive resection margins (OR = 1.71; 95% CI (0.95–3.09); *p* = 0.07) [12]. The meta-analysis by Guan et al. included nine studies with 345 cases in the robotic group and 396 cases in the laparoscopic group for analysis of R status. Positive resection margins were noted in 27 cases (7.8%) in the robotic group and in 33 cases (8.3%) in the laparoscopic group (OR = 1.03; 95% CI (0.41–2.55); *p* = 0.95) [13]. In the pooled analysis of minor liver resections by Wang et al., R0 resection was achieved in 167 (96.0%) of 174 robotic resections and 181 (95.3%) of 190 laparoscopic resections (OR = 1.36; 95% CI (0.48 to 3.83); *p* = 0.56) [4]. However, it should be noted that some studies did not differentiate between malignant and benign cases when reporting the rates of positive resection margins, so the percentages were reported from the entire cohort [9,14]. These numbers were used in some meta-analyses without further differentiation [11,12,13]. In many studies, the number of cases in the laparoscopic liver surgery group is higher than in the robotic liver surgery group due to the earlier adoption of the laparoscopic approach. If no attention is paid to the precise differentiation of malignant and benign cases when interpreting the R0 or R1 rates, this can lead to a lower percentage of positive resection margins.

Furthermore, our meta-analysis showed that significantly larger liver lesions were resected with the robot procedure compared to the laparoscopic procedure (MD = 6.92; 95% CI (2.93–10.91); *p* = 0.0007). This finding was consistent with the results of previous meta-analyses [3,42]. Hu et al. were able to demonstrate significantly larger tumor size in the robotic group based on the data from five studies [3]. Zhang et al. compared the tumor sizes of 743 cases in the robotic group and 1,132 cases in the laparoscopic group in their meta-analysis. In this study, tumor size was significantly larger in the robotic group (WMD = 0.36; 95% CI (0.16–0.56); *p* < 0.001) [42].

Higher freedom of movement, stable three-dimensional visualization, the possibility of using a third arm and the absence of a physiological tremor are the advantages of robotic over conventional laparoscopic surgery. These advantages of robotics enable us to operate safely and precisely in the tight areas and difficult-to-access localizations of the liver [21,22,23]. One of the modern approaches in robot-assisted liver resection is image-guided surgery. Intraoperative navigation can be facilitated using augmented reality during robotic liver surgery. Based on the information from the preoperative and/or intraoperative imaging, 3D reconstructions of the liver can be created in which tumor, intrahepatic bile and vascular structures can be visualized and marked in color. In this way, the operator can better orientate himself/herself during the parenchyma dissection using these virtual landmarks [43]. In addition to the safety distance, a constant dissection of the parenchyma and not leaving the previously defined resection plane are important factors in achieving an R0 resection. Due to the advantages of robotics mentioned above, these properties could be better ensured by the robot. All of these factors may also have contributed to surgeons daring to operate on larger lesions robotically than laparoscopically.

Perhaps the most discussed limitation of the robot-assisted approach is the lack of haptics when compared to standard laparoscopy. As mentioned above, many robotics surgeons believe that the above-mentioned advantages, combined with visual cues, render this theoretical deficiency moot. This review is limited by the fact that tumor location was not taken into account. Future studies need to report tumor location in the posterior or anterior segments and proximity to major hepatic blood vessels to more accurately compare these two approaches. Another confounding factor is the possibility that many robotic surgeons have long experience with laparoscopic liver surgery prior to embarking on robotic-assisted liver resections. The relevance of a minimally invasive surgeon’s previous surgical experience has been highlighted by a recent publication that discusses the initiation, standardization and proficiency phases of the learning curve according to where along the IDEAL (Idea, Development, Exploration, Assessment and Long-term) framework surgeons fall [44].

## 5. Conclusions

With regard to the resection margin status, no significant difference between the robotic and laparoscopic procedures could be determined in the pooled analysis. Tumor size was significantly larger in the robotic group. However, due to the limitations of the published data, randomized controlled trials are needed to truly delineate any potential benefits of robotics in liver resection.

## Figures and Tables

**Table 1:** Characteristics of the included studies.

Author	Year	Country	Study Design	Approach	Cases (n)	Malignant Cases (n)	Positive RM (n)	Sex (m/f)	Study Quality (NOS)
Berber [31]	2010	USA	RCS	RLR	9	9	0	7/2	9
				LLR	23	23	0	12/11	
Troisi [9]	2013	Belgium/Italy	RCS	RLR	40	28	3	27/13	7
				LLR	223	134	12	98/125	
Spampinato [32]	2014	Italy	RCS	RLR	25	17	0	13/12	8
				LLR	25	23	2	10/15	
Croner [33]	2016	Germany	RCS	RLR	10	10	0	8/2	9
				LLR	19	15	0	13/6	
Lai [34]	2016	China	RCS	RLR	100	100	4	66/29	7
				LLR	35	35	3	26/9	
Lee [10]	2016	China	RCS	RLR	70	52	1	46/24	9
				LLR	66	57	1	39/27	
Magistri [35]	2017	Italy	RCS	RLR	22	22	1	18/4	9
				LLR	24	24	1	15/9	
Fruscione [36]	2019	USA	RCS	RLR	57	37	3	20/37	7
				LLR	116	54	4	52/64	
Hu [37]	2019	China	RCS	RLR	58	36	0	33/25	9
				LLR	54	26	0	26/28	
Lim [38]	2019	France/Italy	RCS	RLR	61	61	7	41/20	8
				LLR	111	111	17	83/28	
Marino [39]	2019	Italy	RCS	RLR	14	12	1	8/6	8
				LLR	20	20	3	11/9	
Mejia (a) [30]	2020	USA	RCS	RLR	35	22	2	16/19	8
				LLR	85	32	3	36/49	
Mejia (b) [30]	2020	USA	RCS	RLR	8	7	0	4/4	8
				LLR	13	4	1	6/7	
Cai [40]	2021	China	RCS	RLR	25	12	0	12/13	9
				LLR	27	15	0	18/9	
Lorenz [41]	2021	Germany	RCS	RLR	44	32	2	24/20	8
				LLR	111	58	7	50/61	

LLR = laparoscopic liver resection, NOS = Newcastle–Ottawa scale, RCS = retrospective cohort study, RLR = robotic liver resection, RM = resection margin.

**Table 2:** Summary of the meta-analysis for robotic versus laparoscopic liver resections.

Outcomes	Studies	Cases (n)	OR/MD	95% CI	*p*-Value	Heterogeneity		
	(n)	RLR/LLR				I^2^ (%)	*p*-Value	Model
Positive resection margin	14	457/631	0.71	0.42–1.18	0.18	0	0.98	FE
Operation time	13	565/894	28.12	3.66–52.57	0.02	90	<0.00001	RE
Intra-operative blood loss	11	404/748	−8.56	−70.86–53.73	0.79	82	<0.00001	RE
Length of stay	11	531/846	−0.02	−0.56–0.53	0.94	76	<0.00001	RE
Tumor size	10	433/557	6.92	2.93–10.91	0.0007	52	0.02	RE
Overall complications	13	534/841	0.78	0.56–1.09	0.15	21	0.23	FE
Severe complications	8	284/492	0.92	0.51–1.68	0.79	2	0.42	FE
Conversion	10	426/622	0.74	0.44–1.23	0.25	44	0.07	FE

CI = confidence interval, FE = fixed effects model, LLR = laparoscopic liver resection, MD = mean difference, OR = odds ratio, RE = random effects model, RLR = robotic liver resection.

**Table 3:** Liver malignancies.

Author	Year	Approach	HCC	CCA	CRLM	Other Malignancies
Berber [31]	2010	RLR	3	1	4	1
		LLR	7	0	14	2
Troisi [9]	2013	RLR	3	1	24	0
		LLR	9	2	108	15
Spampinato [32]	2014	RLR	2	2	11	2
		LLR	1	3	16	3
Croner [33]	2016	RLR	4	1	5	0
		LLR	5	2	5	3
Lai [34]	2016	RLR	100	0	0	0
		LLR	35	0	0	0
Lee [10]	2016	RLR	40	3	8	1
		LLR	41	1	13	2
Magistri [35]	2017	RLR	22	0	0	0
		LLR	24	0	0	0
Fruscione [36]	2019	RLR	4	7	uc	uc
		LLR	16	7	uc	uc
Hu [37]	2019	RLR	25	4	2	5
		LLR	23	1	2	0
Lim [38]	2019	RLR	42	2	15	2
		LLR	72	6	23	10
Marino [39]	2019	RLR	4	0	8	0
		LLR	7	0	13	0
Mejia (a) [30]	2020	RLR	18	1	2	1
		LLR	26	0	6	0
Mejia (b) [30]	2020	RLR	4	2	1	0
		LLR	4	0	1	0
Cai [40]	2021	RLR	8	3	0	1
		LLR	9	5	uc	uc
Lorenz [41]	2021	RLR	13	5	12	2
		LLR	33	4	12	9

CCA = cholangiocarcinoma, CRLM = colorectal liver metastasis, HCC = hepatocellular carcinoma, LLR = laparoscopic liver resection, RLR = robotic liver resection, uc = unclear.

## Data Availability

All relevant data are provided in the manuscript.
